# Mouse positioning device for blood brain barrier FUS exposure

**DOI:** 10.1186/2050-5736-3-S1-P20

**Published:** 2015-06-30

**Authors:** Shawn Gong, Bruno Adamczyk, Cleiton Dos Santos, Thiabault Estrade, Laura Curiel

**Affiliations:** 1 Thunder Bay Regional Research Institute, Thunder Bay, Canada

## Background/introduction

Gaucher’s disease is an inherited metabolic disorder that results in a deficiency of the enzyme glucocerebrosidase, which acts on the glycolipid glucocerebroside. This causes a rapid accumulation of glucocerebroside in affected persons, which manifests in organ malfunctions and disfiguration, swelling, and severe neurologic complications, among other symptoms. Enzyme replacement therapy can manage the symptoms but when the brain is affected, the treatment is not effective since the enzymes cannot cross the blood brain barrier (BBB). Imaging of enzyme replacement therapy has shown that only insignificant amounts reach the brain.[1] In this work, we intend to evaluate brain uptake of enzyme replacement after BBB opening mediated by focused ultrasound (FUS) to validate this delivery technique for the treatment of neurologic complications caused by Gaucher’s disease. Our goal is to develop a system for Blood Brain Barrier delivery that can be followed up using positron emission tomography (PET) imaging. It will then be used to create and validate the delivery after intravenous injection of radiolabelled enzymes.

## Methods

**Figure 1:**
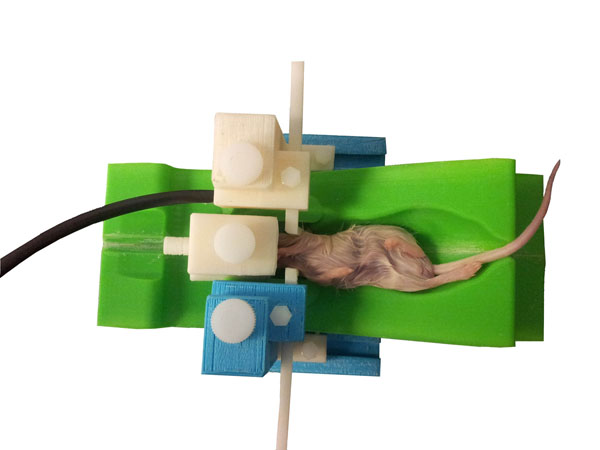
The physical appearance of the stereotactic system holding a test mouse

A stereotactic animal setting for mice was designed using SolidWorks to allow for repetitive FUS exposures of the brain, and was formed with ABS plastic using a MakerBot Replicator 3-D printer. The device was designed for dual MRI and PET imaging after exposures. A waterproof RF coil was attached and formed into the positioner to provide MR images. The device can deliver gas anesthesia and allows for the animal to receive FUS exposures in a repetitive manner. The device has an integrated simple immobilizing system for test mice that allows for experiments to be done at the same location (Figure 1). The system was tested by placing multiple mice one at a time, as well as repositioning the same animal by different operators, and comparing the images produced by the MRI. Images were performed and the deviation of anatomical markers between different experiments was measured. The ability of the positioning system to target the FUS transducer at the same location by the use of the device was measured as well on MR images.

## Results and conclusions

**Figure 2:**
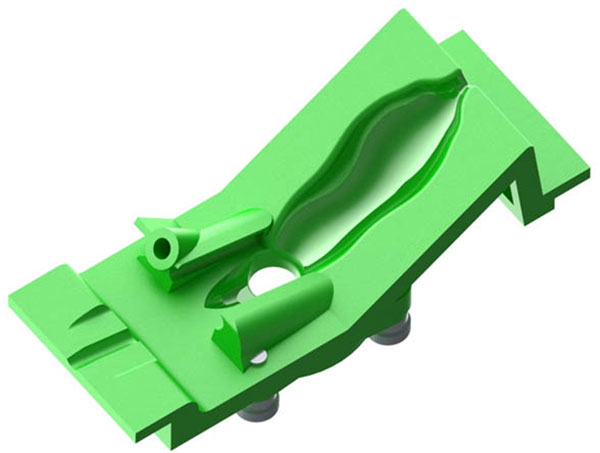
The animal setting that holds the test mouse in the desired position

**Figure 3:**
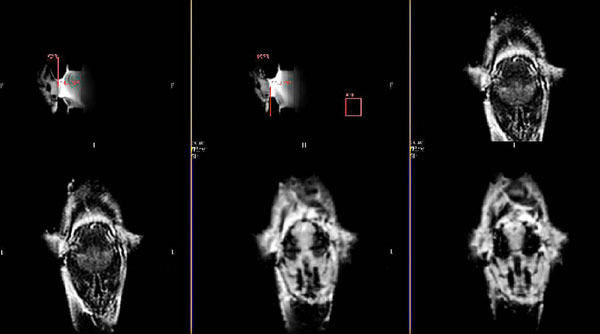
MR images of the brains of two separate mice held using the stereotactic system. As shown the brains are at the same locations

The device was able to hold the test subject in the desired position (Figure 2), and was able to ensure that the water level of the positioning table did not rise above the head of the test subjects thanks to markers in the device. MR images of the device with a test mouse are shown in Figure 3. These images were performed using the coil embedded into the device and allowed for precise targeting by localizing both the FUS transducer and the brain in a repetitive and reproducible fashion. The results indicate that the system is able to hold a mouse in a repetitive position, regardless of the operator. Thus, allowing for much faster focused ultrasound tests that can be seaminglessly followed by PET scans. The ultimate goal will be to perform FUS exposures at a fixed target location, without using the MRI for guidance. We will be then able to perform a PET scan immediately after FUS exposure and validate the delivery of enzymes through the BBB.

**Figure 4:**
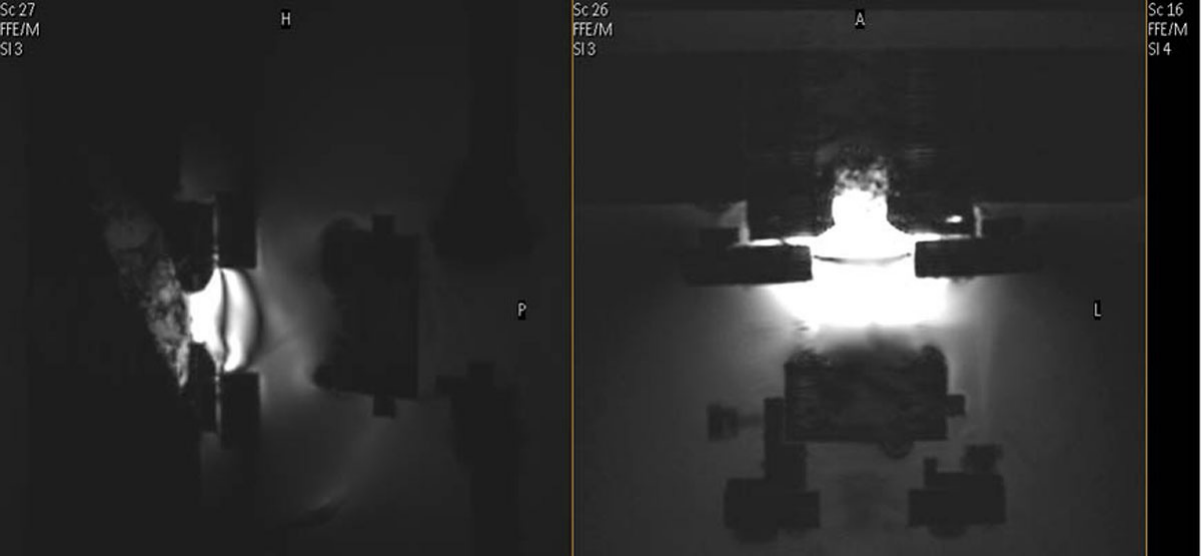
MR images showing the transducer used during trials

## References

[B1] Phenix P Christopher Imaging of enzyme replacement therapy using PET Proceedings of the National Academy of Sciences 2010 107 24 10842 10847 10.1073/pnas.1003247107 PMC2890769 20534487

